# Child Care and Development in the Migration Context of Brazil: A Convergent Parallel Mixed Methods Study at an International Border

**DOI:** 10.1111/cch.70094

**Published:** 2025-05-20

**Authors:** Gabriela D. M. Casacio, Rosane M. M. Silva, Ana L. Penna, Gabrielle Oliveira, Aisha K. Yousafzai, Débora F. Mello

**Affiliations:** ^1^ Postgraduate Program in Public Health Nursing, School of Nursing, Ribeirao Preto University of Sao Paulo Sao Paulo Brazil; ^2^ Postgraduate Program in Public Health in Border Region State University of Western Parana Foz do Iguazu Parana Brazil; ^3^ Department of Global Health and Population Harvard T.H. Chan School of Public Health Boston Massachusetts USA; ^4^ Department of Education Harvard Graduate School of Education Cambridge Massachusetts USA

**Keywords:** child development, childcare, immigration, parenting

## Abstract

**Background:**

Immigrant families in Brazil face challenges such as socio‐economic vulnerability, and limited access to essential services like health and education. These stressors may compromise the capacity of families to provide adequate nurturing care for their young children.

**Methods:**

This study used a convergent parallel mixed methods design to address how the experience of parenting and accessing support among immigrant and refugee caregivers of young children explains their parenting related practices and perceived parenting stress. The study objectives were to (1) describe parental beliefs, care practices and stressors among caregivers of young children living in a migratory context; (2) explore how the environment and social context influence parents' nurturing care for young children; and (3) examine how parents, nurses and educators promote young children's development in a migratory context.

**Results:**

Seventy caregivers participated in the quantitative study, and a subset of 21 caregivers along with 12 nurses and 13 teachers took part in the qualitative study. Quantitative findings revealed that caregivers were not engaging in stimulating activities with their children and perceived their parenting demands to exceed their ability to provide care. These findings were elucidated by the qualitative data, which found that the challenges of migration contributed to parental stress and negative caregiving practices, reduced caregiver–child interaction and was perceived to impact children's health and development.

**Conclusions:**

Understanding these factors may inform interventions to mitigate challenges and offer adequate support for children to thrive in a migratory context.

**Summary**

- Socio‐economic vulnerability is a primary driver of migration, presenting multiple challenges that significantly affect children's health.
- Migration increases parental stress, negatively influencing caregiving practices, reducing parent–child interaction and ultimately impacting children's health and development.
- Discrimination and lack of support compromise childcare quality and hinder immigrant families' adaptation to their new country.
- Comprehensive support fosters stability and provides a strong foundation for effectively nurturing children.

## 1 Introduction

The global immigrant population reached 281 million in 2020 (3.6% of the world's population), comprising 28 million immigrant children (10.1% of the global child population) (International Organization for Migration 2024). Several factors, including language adaptation, family separation, discrimination and low socio‐economic status are associated with immigrants' fears and insecurity (Muggli et al. 2021). Additionally, immigration is linked to lower cognitive, language, motor and social–emotional development in children, which affects future literacy, academic achievement, employment and socio‐economic status (Barajas‐Gonzalez et al. 2022).

From conception to early childhood, a child's brain demonstrates high plasticity and responsiveness, constituting a sensitive period for developing essential skills vital for children's growth and learning through interactions shaped by genetics, environment and experience (Nelson et al. 2024; WHO, UNICEF, WBG 2018). Supporting early child development (ECD) outcomes encompasses five critical components: health, nutrition, responsive caregiving, safety and security and early learning (McCoy et al. 2022).

In contrast, children exposed to prolonged biological and psychosocial stress without adequate adult support may develop maladaptive stress responses, disrupting brain architecture and organ systems (Nelson et al. 2024). Young children in fragile and humanitarian settings are particularly vulnerable, often facing inadequate care. Supporting displaced children and families is imperative to foster healthy development across their lifespan (UNICEF 2024).

In 2022, Brazil recorded 1.6 million official requests for residence and refugee status, including 32 967 immigrant children; this figure does not include undocumented immigrants. Brazilian law mandates that all immigrants have access to essential services, including health, education, work opportunities, residency and citizenship. The Unified Social Assistance System currently supports 236 297 immigrant households living in poverty (Cavalcanti et al. 2023). Evidence suggests that immigrant children in Brazil face discrimination and challenges in accessing critical services like education and healthcare (Vargas et al. 2023).

The cumulative impact of immigration‐related stress requires further research identifying specific contextual stressors and their impact on immigrant parenting, particularly for Latino families (Fuentes‐Balderrama et al. 2023). Despite the evidence regarding stress factors in this context, few studies examine how the challenges and opportunities faced by immigrant families can interfere with childcare and development. For this reason, the study aims to (1) describe parental beliefs, care practices and stressors among caregivers of young children living in a migratory context; (2) explore how the environment and social context influence parents' nurturing care for young children; and (3) examine how parents, nurses and educators promote young children's development in a migratory context.

## 2 Methods

### 2.1 Study Design

**FIGURE 1:**
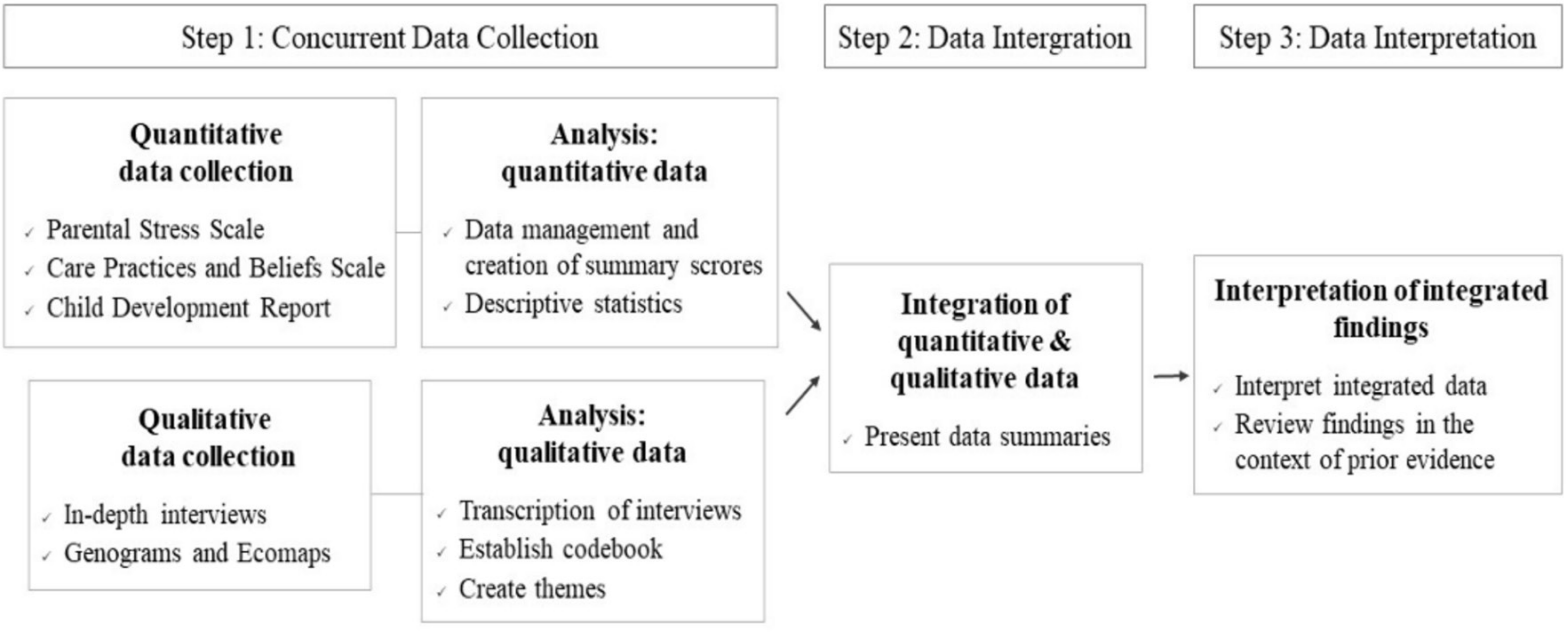
Flowchart of the convergent parallel mixed‐methods design.

A convergent parallel mixed‐methods study was conducted (Figure 1) to deepen our understanding of care for young children in a migratory context in Brazil. In Step 1, quantitative and qualitative data were collected concurrently and analysed independently. In Step 2, the data were integrated to gain additional insights into caregiving for young children in migratory settings. Finally, in Step 3, the integrated findings were discussed (Halcomb and Hickman 2015).

### 2.2 Study Setting

This study was conducted in Foz do Iguacu, Brazil, a city on the triple border with Argentina and Paraguay, with a population of 285 415 (IBGE 2024), including 16 303 immigrants (5.7% of residents), surpassing the global average of 3.6% (IBGE 2024; United Nations 2024). Approximately 9180 local immigrants (54.1%) are registered for social assistance programmes and identified as low‐income families (Foz do Iguaçu 2024). This demographic includes refugees and legal immigrants originating from Argentina, Bangladesh, Bolivia, Colombia, Cuba, Ecuador, Egypt, Haiti, Lebanon, Paraguay, South Africa, Syria and Venezuela.

Immigrants to Brazil are motivated by the need to escape violence and poverty and to seek improved living conditions and opportunities for work and study. National legislation guarantees immigrant access to education, healthcare (*Sistema Unico de Saude* [*SUS*]), and social assistance services, including cash transfer programmes like Bolsa Família (IOM 2022).

### 2.3 Study Selection and Eligibility

A convenience sampling method recruited immigrant parents using children's records from the Municipal Early Childhood Education Center, primary healthcare centres and social assistance institutions. Caregivers were eligible if they were over 18, responsible for children aged 6 or under and residing in Foz do Iguacu for at least 3 months. Exclusion criteria included caregivers with mental health diagnoses and special health needs or those institutionalized, hospitalized or caring for children with special healthcare needs.

For the qualitative study, we recruited three stakeholder groups: (i) caregivers (using the same inclusion criteria as the quantitative study), (ii) primary healthcare nurses with at least three months of experience and (iii) teachers from the Municipal Early Childhood Education Center with at least 3 months at the institution. Families meeting the inclusion criteria were contacted through teachers from the Municipal Early Childhood Education Center and Community Health Workers (CHW), and face‐to‐face interviews were scheduled by telephone. During these interviews, families were invited to participate in both the quantitative and qualitative components of the mixed‐methods study. All caregivers in the qualitative study also participated in the quantitative study. Sample size calculation was not possible due to the lack of detailed data on immigrant families in the municipality's information system, which does not include specific information on the number of children from immigrant families.

### 2.4 Data Collection

Data collection was conducted by the first author between April and October 2023, who holds a master's in public health and is pursuing a PhD in the same field, with specialized training in the research instruments used. Residing in the triple border area, the author is familiar with the local culture and context. The co‐authors hold doctoral degrees and possess extensive expertise in child development and parenting, particularly within the Brazil context.

Participants were informed about the research and, after agreeing to participate, signed the consent form (freely and with information about the study). All interviews were conducted in‐person, at home, with caregivers, and for nurses and teachers, meetings were conducted in a private space at their workplace. Field notes were made about impressions of the family and professional environment.

All data collection was conducted in Portuguese. The first author, fluent in English and proficient in Spanish, initially offered a translator, but the participants, who spoke Portuguese with some Spanish mixed in, did not require translation services. The first author conducted the interviews in Portuguese and translated the quotes into English, ensuring an accurate representation of the participants' responses. No interviews were repeated.

#### 2.4.1 Quantitative Component

For each caregiver, three questionnaires, previously validated in Brazil, were administered: Parenting Beliefs and Caring Practices Scale (Martins et al. 2010), Parental Stress Scale (Brito and Faro 2017) and a child development screener from the Monitoring Child Development in the Integrated Management of Childhood Illness (IMCI) programme (PAHO 2012).

The first questionnaire is based on Keller's theoretical model (Keller 2002) and examines parental practices, beliefs and values within various cultural contexts. It consists of two subscales (practices performed and importance attributed to practices) with 18 identical items across two dimensions: primary care (primary care and body contact systems) and stimulation (body stimulation, object stimulation and face‐to‐face contact systems). The scale includes domains such as primary care (e.g., comforting when crying, feeding and ensuring cleanliness), physical contact (e.g., holding, hugging and safety), physical stimulation (e.g., tickling, massaging and physical activities), object stimulation (e.g., providing toys, playing games and showing interesting things) and face‐to‐face Interaction (e.g., talking, explaining, listening and eye contact).

The second questionnaire, the Parental Stress Scale, assesses the stress levels experienced by primary caregivers due to their parental role. Comprising 16 items across two factors (parental stressors and parental satisfaction), higher scores indicate greater stress. Both scales use a 5‐point Likert scale: 0 = *strongly disagree*, 1 = *disagree*, 2 = *undecided*, 3 = *agree* and 4 = *strongly agree*. The reliability of both scales was confirmed with Cronbach's alpha of 0.97 for each.

Lastly, the third questionnaire was a child development monitoring tool for children in the first 6 years of life designed for professionals in the primary healthcare system (PAHO 2012).

#### 2.4.2 Qualitative Component

Following completion of the quantitative questionnaires, in the same meeting, caregivers were invited to participate in the qualitative study. For those who accepted, a semi‐structured interview was administered. Separately, appointments with nurses and teachers for individual interviews were made. Interviews were reviewed regularly throughout the data collection period and stopped when data reached saturation (i.e., no new information was heard).

The interview guides were developed by the first and senior authors including questions about early childhood development, care practices and beliefs and parental stress, emphasizing the cross‐cultural migratory context. In addition to the in‐depth interview, caregivers completed two types of family and social network maps: (1) a **genogram**, which is a detailed, visual representation of family relationships, histories and dynamics across multiple generations and includes information about family members' roles, significant life events, and intergenerational patterns, helping to uncover the emotional, social and relational aspects of family life; and (2) an **ecomap**, which is a diagram that illustrates the individual's relationships and interactions with their broader social environment, including family, friends, work and community support systems. The ecomap helps identify the strengths and challenges within these relationships, highlighting both sources of support and potential stressors in the caregiver's social network.

All interviews were audio‐recorded and transcribed verbatim in Portuguese. To ensure content validity, four transcripts were returned to participants for feedback, and all confirmed their accuracy.

### 2.5 Data Analysis

Quantitative data were exported to Excel for descriptive statistical analysis, with scales analysed according to the authors' criteria. For the Parenting Beliefs and Caring Practices Scale, scores were categorized as below average (32–34 points), average (35–37 points) and above average (38–40 points). In the Parental Stress Scale, responses were stratified to calculate the absolute and relative frequency of each item.

An inductive thematic analysis approach was employed for the analysis of the qualitative data, which was based on Bronfenbrenner's bioecological theory and focused on the proximal context (person, process, context and time) (Bronfenbrenner and Ceci 1994). The interview transcriptions were uploaded to Atlas.ti (Version 24) to help manage interview transcripts through the organization of codes, sub‐codes and themes.

First, the meaning (codes and sub‐codes) of the participants' responses was identified, and a codebook was created. All transcripts were coded by the first author, and a sub‐sample was also coded by an independent researcher to validate the codes. The codebook was updated as new codes emerged, and regular discussions on the evolving ideas from the codes were held within the study team. Following code narrative write‐ups, codes and sub‐codes were compared for similarities and differences and then organized into broader themes (Creswell and Poth 2018). Twelve codes and 30 sub‐codes emerged from the data analysis (Table 1), and thematic narratives were subsequently prepared.

**TABLE 1:** Codes and sub‐codes generated from qualitative analysis.

Codes	Sub‐codes
Care practices	Negative care practices and violence Places and objects Positive attachment and interaction
Challenges	Documental situation Family distancing Language Socio‐economic vulnerability
Child development	Child profile Negative development Positive development
Cultural elements	Cultural inclusion Cultural practices and beliefs
Discrimination	With children With families
Health services	Access, first contact and prevention Longitudinally
Immigration process	Reasons to migrate Path and adaptation Positive factors found in Brazil
Orientation	(Lack of) Orientation to caregiver (Lack of) Families' interest in information (Lack of) Willingness and knowledge to listen and guide
Positive strategies to improve child development	No sub‐codes
Stress	Intergenerational inheritance Intra‐family dynamics Negative stress experienced by the child Perception of insecurity
Support	Educational support Lack of parenting support and resilience Social support network Strong foundation provided by families
Time and adaptation	No sub‐codes

### 2.6 Ethical Considerations

This study received approval from the Research Ethics Committee of Ribeirao Preto Nursing School at the University of Sao Paulo (ethical appreciation number 5.951.090, 2023). Participants were informed about the study's objectives and signed a two‐way Free Informed Consent Form (FICF), with one copy provided to them and another retained by the researcher. The FICF was available in Portuguese, Spanish and English, with an interpreter available if needed; however, none requested this service. Personal identifiers were removed from transcripts, and quotes were anonymized. Any developmental concerns identified during data collection were addressed by encouraging families to seek appropriate health services.

## 3 Results

### 3.1 Quantitative Findings

#### 3.1.1 Demographic Characteristics

Seventy caregivers participated in the quantitative study, whereas 12 declined due to concerns about identification as asylum seekers, potential loss of social assistance (cash transfers) or safety issues stemming from their flight from violent environments. Most caregivers were from Venezuela (44%), Paraguay (29%) and Colombia (9%). The majority immigrated for better living conditions (60%), whereas smaller percentages were refugees (13%) or seeking study opportunities (14%). Approximately one‐third had lived in Foz do Iguacu for 1–3 years (33%), whereas just over one‐third had resided there for 4–9 years (39%). Most primary caregivers were not in paid employment (66%). The majority of families lived in houses (84%) and had access to basic sanitation (94%), with half receiving cash transfers from the Bolsa Família programme (51%). Most primary caregivers lived with a partner (67%) and had one (43%) or two children (30%), with 40% born in Brazil and 37% in Paraguay. Nearly all children were attending school (91%) (Table 2).

**TABLE 2:** Socio‐demographic and characteristics of principal caregivers and children (*n* = 70).

Variables	Frequency *n* (%)
Parental relationship with young child	
Mother	64 (91%)
Father	5 (7%)
Grandmother	1 (1%)
Caregiver's age (years)	
16–22	8 (11%)
23–29	24 (34%)
30–40	27 (39%)
41–50	11 (16%)
Caregiver's education level	
Elementary school	11 (16%)
High school	32 (46%)
Undergraduate degree	27 (39%)
Family country of origin	
Venezuela	31 (44%)
Paraguay	20 (29%)
Colombia	6 (9%)
Argentina	4 (6%)
Haiti	3 (4%)
Cuba	2 (3%)
Syria	1 (1%)
Senegal	1 (1%)
Ecuador	1 (1%)
Bolivia	1 (1%)
Reason to immigrate	
Better living conditions	42 (60%)
Refuge	9 (13%)
Study	10 (14%)
Work	7 (10%)
Health treatment	2 (3%)
Duration of time living in Brazil	
3–11 months	12
1–3 years	23
4–9 years	27
> 10 years	8
Caregiver in paid work	
Yes	24 (34%)
No	46 (66%)
Type of residence	
House	59 (84%)
Apartment	5 (7%)
Slum	4 (6%)
Shelter	1 (1%)
Hotel room	1 (1%)
Access to basic sanitation	
Yes	66 (94%)
No	4 (6%)
Cash assistance	
Yes	36 (51%)
No	34 (49%)
Live with a partner	
Yes	47 (67%)
No	23 (33%)
Number of children	
1	30 (43%)
2	21 (30%)
3	14 (20%)
> 3	5 (7%)
Child nationality	
Brazil	55 (40%)
Paraguay	51 (37%)
Venezuela	20 (15%)
Argentina	4 (3%)
Colombia	3 (2%)
Haiti	1 (1%)
Cuba	2 (1%)
Ecuador	1 (1%)
Child in school	
Yes	64 (91%)
No	6 (9%)

##### 3.1.1.1 Parenting Beliefs and Caring Practices Scale

The Parenting Beliefs and Caring Practices Scale revealed that most caregivers engaged in practices related to primary care (96%), body contact (85%) and face‐to‐face contact (96%). Fewer caregivers practiced tasks linked to body stimulation (54%) and stimulation with objects (69%) (Table 3). Responses on the Likert scale were categorized into three classes: above average (never and almost never), average (not sure) and below average (almost always and always).

**TABLE 3:** Frequency of responses for the Parental Beliefs and Care Practices Scale (*n* = 70).

Parental Beliefs and Care Practices Scale	Practices *n* (%)	Importance to practice *n* (%)
Primary care		
Above average	67.0 (96%)	67.6 (97%)
Average	2.6 (4%)	1.6 (2%)
Under average	0.4 (1%)	0.8 (1%)
Body contact		
Above average	59.6 (85%)	60.6 (87%)
Average	7.0 (10%)	6.6 (10%)
Under average	3.3 (5%)	2.6 (4%)
Body stimulation		
Above average	37.6 (54%)	51.6 (74%)
Average	17.3 (25%)	9.0 (13%)
Under average	15.0 (21%)	9.3 (13%)
Stimulation by objects		
Above average	48.2 (69%)	60.0 (86%)
Average	11.5 (16%)	6.5 (9%)
Under average	10.2 (15%)	3.5 (5%)
Face‐to‐face contact		
Above average	67.3 (96%)	68.3 (98%)
Average	2.3 (3%)	1.6 (2%)
Under average	0.3 (0%)	0.0 (0%)

##### 3.1.1.2 Parental Stress Scale

Table 4 presents the absolute and relative frequency of responses from participants on the Parental Stress Scale. Most caregivers reported positive satisfaction with their caregiving role. Almost everyone (97%) agreed they were happy as parents and felt close to their children. Additionally, 96% enjoyed spending time with their children and held an optimistic view of their future after having children. Regarding parental stressors, 66% agreed that caring for their children required more time and energy than they could provide. About one‐third reported that having children left little time and flexibility (30%) and found it challenging to balance responsibilities due to childcare duties (31%). When asked hypothetically if they would make different life choices regarding having children, only 10% of caregivers agreed with this statement (see Table 4).

**TABLE 4:** Frequency of responses for each item of the Parental Stress Scale (*n* = 70).

Frequency *n* (%)					
Item	Strongly disagree	Disagree	Not sure	Agree	Strongly agree
1 I am happy in my role as a parent	0	1 (1%)	1 (1%)	10 (15%)	58 (83%)
2 Caring for my child(ren) sometimes takes more time and energy than I have to give	9 (13%)	11 (16%)	4 (6%)	23 (33%)	23 (33%)
3 I feel close to my child(ren)	0	0	0	17 (24%)	53 (76%)
4 I enjoy spending time with my child(ren)	0	0	3 (4%)	13 (19%)	54 (77%)
5 My child is an important source of affection for me	0	0	0	7 (10%)	63 (90%)
6 Having children gives me a more optimistic view of the future	1 (1%)	0	2 (3%)	11 (16%)	56 (80%)
7 The main source of stress in my life is my child	33 (47%)	20 (29%)	5 (7%)	3 (4%)	9 (13%)
8 Having children leaves little time and flexibility in my life	16 (23%)	29 (41%)	4 (6%)	9 (13%)	12 (17%)
9 Having children has been a financial burden	23 (33%)	24 (34%)	4 (6%)	9 (13%)	10 (14%)
10 It is difficult to balance different responsibilities because of my child	21 (30%)	20 (29%)	7 (10%)	14 (20%)	8 (11%)
11 My child's behaviour is often embarrassing or stressful for me	46 (66%)	13 (19%)	5 (7%)	5 (7%)	1 (1%)
12 If I had to do it all over again, I might decide not to have children	51 (73%)	11 (16%)	1 (1%)	4 (6%)	3 (4%)
13 I feel overwhelmed by the responsibility of being a parent	33 (47%)	21 (30%)	7 (10%)	6 (9%)	3 (4%)
14 Having children has meant having fewer choices and less control over my life	35 (50%)	18 (26%)	2 (3%)	12 (17%)	3 (4%)
15 I am satisfied as a parent	1 (1%)	3 (4%)	0 (0%)	7 (10%)	59 (84%)
16 I find my children enjoyable	0 (0%)	1 (1%)	1 (1%)	4 (6%)	64 (91%)

Only 21 caregivers allowed their children to undergo a development evaluation. Among the subset assessed (*n* = 30), six children were under 1 year old, six were between 1 and 2 years old, ten were aged 3–4 years, and eight were aged 5–6 years. Eighteen children exhibited typical development, nine showed developmental concerns (not performing at least one age‐appropriate activity), and three were identified with probable developmental delays, warranting further diagnostic evaluation. Caregivers were provided guidance on the importance of early identification of developmental delays and encouraged to schedule appointments at primary healthcare services.

### 3.2 Qualitative Findings

#### 3.2.1 Demographic Characteristics

We interviewed 21 caregivers (Immigrants), 12 nurses (Brazilian) and 13 teachers (Brazilian). Eight caregivers declined to participate in the qualitative study, citing insecurity, whereas no teachers or nurses refused. The average interview duration was 50 min for caregivers and 40 min for service providers. Among the caregivers, 18 were mothers and three were fathers, with an average age of 30. They migrated from Paraguay (*n* = 7), Venezuela (*n* = 7), Colombia (*n* = 5), Bolivia (*n* = 1) and Haiti (*n* = 1). Educational backgrounds included six with primary education, five with secondary education, and ten with undergraduate degrees. Reasons for immigration included seeking better living conditions (*n* = 10), study opportunities (*n* = 7), work opportunities (*n* = 3) and health treatment (*n* = 1). Most caregivers had paid employment (*n* = 13) and received cash transfers due to low income (*n* = 12). Among the nurses, most were female (*n* = 11), with an average age of 34 and 8 years of service. For the teachers, only one was male, with an average age of 40 and 9 years of service.

#### 3.2.2 Inductive Thematic Analysis

Four themes were identified: (i) *challenges in the migration process* perceived to be linked to vulnerability, negative care practices and risks to child health; (ii) *language and documentation barriers* experienced by immigrant families, impacting access to services and support for their children; (iii) *discrimination and lack of support* perceived by immigrant families to exacerbate parental stress, hinder adaptation to a new setting and affect care practices; and (iv) *caregiver support as positive strategies* that enhanced the stability of care and supported healthy child development.

##### 3.2.2.1 Challenges in the Migration Process

Socio‐economic vulnerability and limited access to healthcare and education in their countries of origin were primary factors motivating families to migrate in search of a better life.

We came due to economic problems. We had a bakery, but we could only sell enough to buy more ingredients for new products—there was no money left over. So, we decided to come to Brazil because we did not have anything to eat there. (Caregiver 4)

My daughters did not have a school. The health was also bad; my children were developing infections. (Caregiver 21)

The migration trajectory encompassed numerous challenges, including inadequate accommodations, nutritional deficiencies and bureaucratic delays to acquiring documents.

One student's family walked to Foz do Iguacu barefoot, their feet covered in wounds. They slept wherever possible until they reached the shelter. (Teacher 5)

I stayed at the border for 15 days in some tents. The Brazilian's government helped us with our documents and a free flight. Many people were crossing the border, which was time‐consuming and stressful. I was going to sleep on the street like many [Nationality redacted] do in Brazil, but my cousin's friend welcomed us into his home. The apartment was tiny and with two other families. He got cardboard from the street to put on the floor, and we stayed there for a week. (Caregiver 10)

Even while receiving humanitarian assistance, families faced additional obstacles, impacting children's health.

The Brazilian's government was providing support in the border, called internalization, but there are many rules. So, we had to save money to cross the border. (Caregiver 7)

Crossing the border was challenging. My son developed bronchitis after we spent a night outside in the intense cold. (Caregiver 4)

I stayed at the border for 15 days, waiting for documents and vaccinations. Afterward, we travelled by bus and boat for 5 days to cross the river, then took another bus and hitchhiked. My daughters lost a lot of weight. (Caregiver 21)

Upon their arrival in Brazil, families encountered numerous challenges in adapting to the new environment, primarily stemming from socio‐economic vulnerabilities.

Many immigrants arrive with nothing and go straight to shelters until they find a job and a way to survive independently. (Teacher 5)

Parents bring their children, but they have no job or anywhere to live, which leads to an increase in violence, difficulty in accessing education, healthcare and support. (Nurse 12)

Upon our arrival, the priest housed us for 5 days until we secured this place, which had no ceiling and a roof full of holes. (Caregiver 5)

Arriving in Foz do Iguacu was challenging; my first purchases were a bed and cooking supplies. The lack of money is the toughest part. (Caregiver 2)

Service providers linked these challenges to negative care practices and childhood stress.

The child lived in poor hygiene conditions. Their precarious home in their country of origin prompted their migration, but their current situation is still not ideal. (Nurse 2)

We observe behaviours such as spontaneous crying, fighting and discord among the immigrant children. (Teacher 8)

##### 3.2.2.2 Language and Documentation Barriers

Upon arriving in Brazil, the challenges to regularizing documentation hindered families' access to health services adversely affecting child health.

I've had entire families who did not have documents, and the vaccine is all delayed. (Nurse 2)

They come to the health unit only when it is an emergency, because they know they will not have to present the documentation (Nurse 3).

One of the main adaptation challenges was the difficulty with the new language. Limited health and prevention information in Portuguese increased parental stress and compromised children's health by hindering providers' understanding of families' needs.

We have some informational folders, but they are only available in Portuguese. (Nurse 3)

The first day I went for a consultation was difficult because I did not know how to speak or where to go. People did not understand me. (Caregiver 2)

The migration process even increases parents' stress, because some mothers arrive without knowing anything. (Nurse 4)

Once, a mother reported that everything was fine with her child and had no complaints, but the child appeared very agitated. Upon evaluation, I discovered the child had yeast infection. (Nurse 3)

Language difficulties were also identified as the primary reason for reduced interaction with other children at school and delays in child development. However, when support was offered to families and children, adapting to the new context was less challenging.

The teacher said that she does not like to participate in anything; she is always alone and quiet. (Caregiver 6)

Some children do not understand Portuguese. They suffer a lot and wasn't developing. (Teacher 7)

I could not understand what the Chileans, Haitians and French speakers were saying. However, we managed to communicate using Google Translate. (Nurse 8)

##### 3.2.2.3 Discrimination and Lack of Support

Participants perceived discrimination against immigrant children, which may limit adherence to child development guidelines by nurses and teachers, compromising the quality of childcare for migrant families. Discrimination also hindered their ability to connect with peers and adapt to their new country. Furthermore, a disregard for cultural practices leads to lower quality care that can negatively impact children's health.

We have a certain prejudice; we think they [immigrants] came to take the Foz do Iguacu’ place, to take our money. (Nurse 12)

They [Brazilians] treat her [daughter] as if she were abnormal, as if she were not part of this group. Other children do not come near her. (Caregiver 1)

The daycare only talks about my daughter and how she behaves when I ask. No one has ever told me how to stimulate her at home. (Caregiver 17)

Most of my friends here are Spanish speakers, as I've struggled to connect with Brazilians, who seem uninterested in friendship. (Caregiver 1)

We follow the traditions of our culture [Brazil]. We understand them [immigrants] as minorities; we do not know enough to include their culture. (Teacher 1)

I have difficulty entering the house [home visits], so I will not go to the house and do any action there. (Nurse 1)

The distance from extended families represents another significant barrier that compromises adaptation and contributes to negative caregiving practices.

The hardest part is not having a [extended] family and having to face this process alone. (Caregiver 8)

My son goes to college with me every day, because I do not have a trusted person to stay with him. (Caregiver 12)

I teach them to be independent, because it's just me and them. So, the girl [5 years old] takes a shower alone, puts on clothes, clean the house and sleep alone. (Caregiver 20)

When they get home, they give the phone to the child, who spends the entire day and night on it. The child goes to bed late and arrives here tired. The children tell us this. (Teacher 10)

**FIGURE 2:**
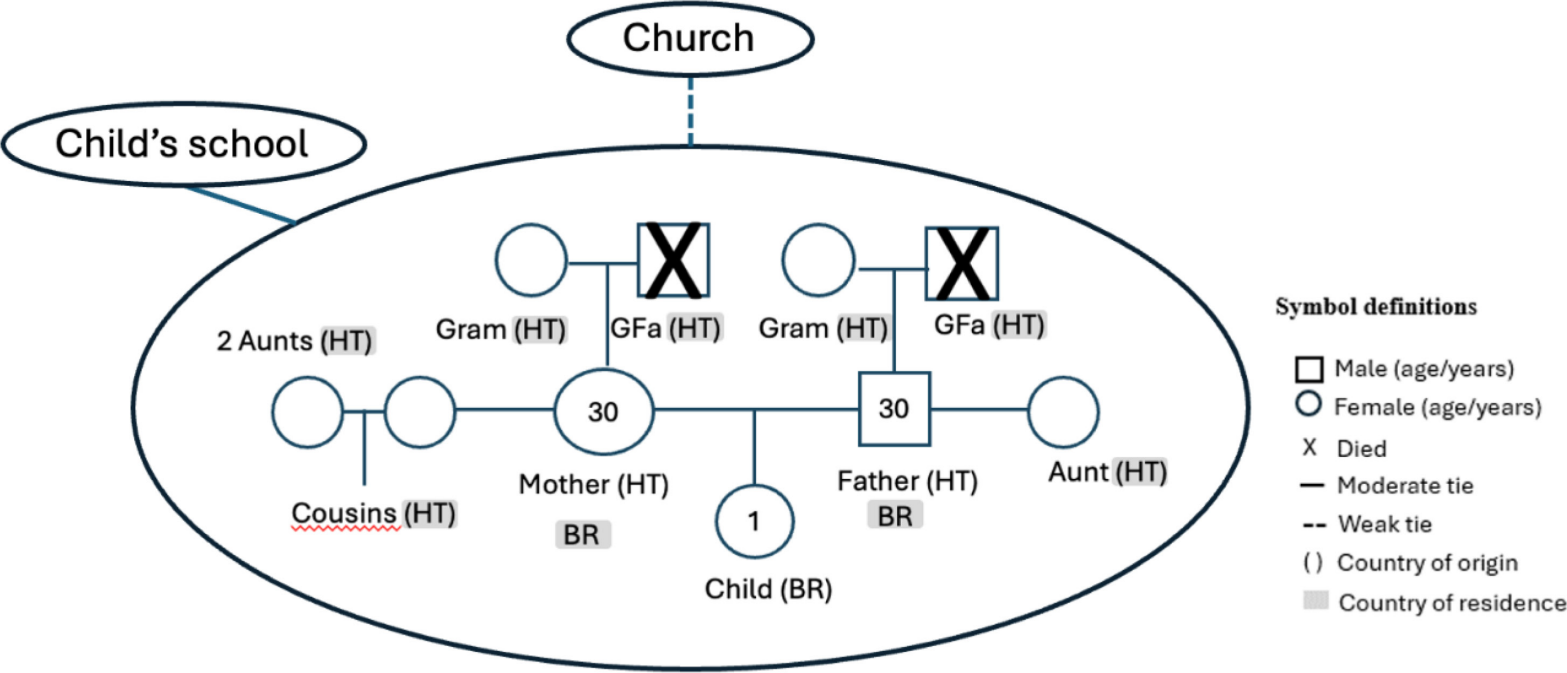
Genogram and ecomap: illustrating the family tree and social context (Caregiver 11). BR, Brazil; GFa, grandfather; Gram, grandmother; HT, Haiti.

The use of genograms and ecomaps illustrated these findings by depicting family relationships and social contexts, revealing the significant lack of social support among migrants (Figure 2).

All reported issues heighten parental stress and reduce interaction time with children. Caregivers expressed feeling exhausted and overwhelmed by daily challenges, leading to negative practices. Despite these obstacles, they remained committed to nurturing positive relationships and prioritized quality time whenever possible while facing constraints that hindered their opportunities to thrive and improve their quality of life.

Every morning is a new challenge, and we always struggle to solve it and work on stress. (Caregiver 11)

When I am tired, I start screaming, especially at my son, who cries. (Caregiver 4)

We only have time when he [son] got back from daycare. But he arrives tired and wanting to sleep, so quality time is just half an hour per day. (Caregiver 9)

One of the reasons I have not graduated yet is because I do not have enough time. When I do have free time, I choose to spend it with her [daughter] despite feeling exhausted and so tired. (Caregiver 1)

##### 3.2.2.4 Caregiver Support as Positive Strategies

The education system was identified as a key support for families and children, particularly in developing language skills, facilitating peer interaction and promoting child health. When teachers understood children's needs and integrated their culture into daily practices, it fostered a sense of belonging.

My son had some problems at school due to language issues. But they are helping a lot with us. (Caregiver 21)

A family said they lacked healthcare access, but we secured an appointment for their child. (Teacher 10)

I did a project on [Nationality redacted] culture, dressing a student in a traditional outfit and performing a choreography. This made the student, and her family feel welcomed and happy.

Creating welcoming spaces for immigrant families and providing training for teachers and health professionals in Spanish—the most spoken language in this region—were emphasized as essential for effective communication and addressing children's needs. Comprehensive support enables the child to receive nurturing care.

There should have been someone available to communicate with them, provide guidance, establish a bond of trust and offer direction. (Nurse 9)

I have a student with immigrant parents from diverse backgrounds who have good jobs, and this child interacts and learns more easily. (Teacher 11)

Now I can guarantee food for my daughters, a paediatrician. In my country, it wasn't possible. (Caregiver 21)

Although families in Brazil received healthcare, education and cash transfers, caregivers reported that these provisions were insufficient to meet their needs.

We are in a good place to take care of him, we have a home, a paediatrician. (Caregiver 10)

I'm happy because I find medicine at the health centre, and I get free milk. But I need to buy food and it's expensive, what I receive from Bolsa Familia [cash transfer] is not enough. (Caregiver 2)

Here in Foz do Iguacu, children only have a 4‐h day at school, and they have no other activities such as library and playground. (Caregiver 12)

## 4 Discussion

In this convergent parallel mixed‐methods study, the quantitative analysis revealed that although caregivers recognize the importance of engaging in stimulating activities with their children, most do not consistently participate in these practices. However, caregiving behaviours such as primary care, body contact and face‐to‐face interaction were positive. Caregivers reported that parenting demands limit their time and flexibility, making it challenging to manage multiple responsibilities. Additionally, the assessment identified developmental concerns in nearly half of the children.

The qualitative component corroborated the quantitative finding that although parents were satisfied with their caregiving role, they were challenged by the demands of this role and managing their responsibilities. However, the qualitative findings also expanded on this finding and provided insights with regard to revealing that challenges within the migration context contribute to parental stress and negative caregiving practices, diminishing caregiver–child interaction and negatively affecting children's health and development. Some challenges may be more acutely felt at different stages in the migratory journey. Financial hardship and food insecurity drove families to seek better living conditions in Brazil, where access to healthcare, education and social security are universal rights for all immigrants (IOM 2022). Despite Brazilian's provision of humanitarian assistance, children in transit remain particularly vulnerable to health risks and human rights violations, as supported by prior research (Lira et al. 2022; Naranjo et al. 2023; de Oliveira 2021; Vargas et al. 2023).

The vulnerable conditions upon arrival in Brazil (illness, homelessness, lack of support and language barriers) are associated with stress and negative caregiving practices, aligned with the literature (Andrade et al. 2023; Arakelyan and Ager 2021). Similar to our findings, delays in processing documentation reduce access to health services and compromise childcare, as highlighted in a study conducted in Brazil (Cavalcante et al. 2022).

The quantitative descriptive data indicated that most caregivers in the study were in low‐paid employment and also received social protection cash transfers. The qualitative findings explained how caregivers identified their socio‐economic vulnerabilities as the primary barrier to settlement, restricting their children's learning environment. Literature indicates that children from low‐income families lack access to toys and stimulating environments that support development and learning (Ogletree et al. 2020; Rothstein et al. 2021), a finding echoed in our study. Exposure to economic and social stressors also increased caregiver burden, poorer parental health and heightened family conflict (Sim et al. 2023). Despite these adversities, a study involving refugee highlighted positive parent–child interactions (Sim et al. 2023). Both perspectives align with our investigation, as we observed positive and supportive parent–child interactions alongside significant parental stress.

Compounding socio‐economic vulnerabilities, language barriers were identified as a significant obstacle to accessing health services and receiving adequate guidance for children's health and care, particularly due to the lack of bilingual staff. Studies consistently show that this compromises care quality through poor communication, strained relationships and misunderstandings, undermining the delivery of care and support and exacerbating stress levels among both parents and children (Gerchow et al. 2021; Lebano et al. 2020). Additionally, language barriers are associated with reduced interaction between parents and teachers, as well as child segregation, findings supported by literature (Buttiler et al. 2023; He and Ge 2024).

A unique insight pertaining to barriers faced by families that emerged from the qualitative interviews with caregivers was that refugees and immigrants face additional challenges stemming from discrimination, which impacts the mental health of both children and their mothers (Jarvis and Kirmayer 2023; Qu et al. 2021). Our study further elucidates that discrimination, language barriers and low‐income levels contribute to a lack of guidance and social support, fostering feelings of loneliness and isolation (Lee et al. 2020).

The stress reported by families in the quantitative survey was likely experienced at different levels in our study population as elucidated in our qualitative interviews, which highlighted that family separation intensifies loneliness and stress, hindering adaptation. Further, parents experiencing lower social support, as shown in our ecomaps, exhibit reduced parenting self‐efficacy, particularly when experiencing anxiety and depression symptoms (Fierloos et al. 2023). Our study suggests that family distancing increases loneliness and parental stress, compounded by inadequate social support, which diminishes caregiver–child interaction. We found that lower self‐efficacy and higher parenting stress are linked to increased screen time, consistent with the literature (Mansor et al. 2021; Veldman et al. 2023).

Maternal reports indicate that mothers prioritize childcare over career and educational opportunities, often sacrificing personal growth and economic advancement. Although this is a common finding, the challenges they face in this context can exacerbate the negative impact (Juang et al. 2018; Lee et al. 2020).

The educational system effectively supports children by alleviating challenges and promoting adaptation through language acquisition, guidance and a sense of belonging, similar to findings in rural China (Chen et al. 2020). The qualitative data also enabled analysis of recommendations for reducing barriers as expressed by the study participants. Interviewees emphasized that reducing barriers requires policies and practices that enhance awareness of language and cultural inclusion, align with previous studies (McDevitt 2021; Mitchell et al. 2020; Norheim and Moser 2020). This approach creates safe spaces for children and parents, promoting their well‐being and successful community integration (Andrade et al. 2023).

We found examples of good practices by teachers, such as cultural inclusion, the use of technology to facilitate communication and support with clothing and medical appointments. However, there is a need for training in cultural competencies to better include immigrant children in schools. Effective communication from healthcare and education providers—characterized by cultural sensitivity, empathy and ongoing relationships—is crucial for delivering safe and high‐quality care (Dougherty et al. 2020).

Research indicates that healthcare providers from diverse ethnic backgrounds, trained interpreters, effective care coordination and strong patient–provider communication with follow‐up support are essential for meeting immigrants' needs (Pandey et al. 2021). Integrating health, nutrition, child protection, social protection and education sectors can enhance interactions, foster positive caregiving practices and establish a solid foundation for nurturing children (Little et al. 2021; WHO, UNICEF, WBG 2018).

This study has several limitations. First, the lack of detailed information on the number and location of immigrant families posed challenges for sample calculation and participant recruitment. Second, although interviews were conducted in Portuguese and participants primarily spoke Spanish, the language difference may have influenced engagement and interpretation. Although the first author has some proficiency in Spanish, Portuguese is her primary language, and certain cultural nuances may have been overlooked. Lastly, given that families were fleeing vulnerable situations, some may have hesitated to share information due to fear of harm, potentially leading to the loss of valuable data. However, adopting a mixed‐methods approach permitted expanding learning from the quantitative data and provided depth of information and insights from the participants that chose to participate in the study.

## 5 Conclusion

In conclusion, immigrant families in Brazil face significant challenges within their migratory context. Socio‐economic vulnerabilities, language barriers, cultural differences and inadequate support systems exacerbate parental stress, whereas the need to work and study in pursuit of a better quality of life further limits the time parents can spend with their children. External barriers, including discrimination, social isolation and lack of access to toys and stimulating environments, reduce opportunities for play and learning, which in turn negatively impacts children's health and development. Although caregivers engage in essential caregiving tasks, their limited involvement in activities that promote stimulation, and interaction suggests more constrained beliefs about the broader dimensions of nurturing care. To address these challenges, integrating social protection with health and education sectors—while ensuring cultural sensitivity—can create a more supportive environment for effective parenting, ultimately enabling children to thrive. Future research will be needed to further investigate associations between reasons of immigration and parenting practices that may help better tailor policies and programmes to the heterogenous needs of this population, often in vulnerable situations.

## Author Contributions

**Gabriela D. M. Casacio:** conceptualization, data curation, formal analysis, visualization, writing – original draft, methodology, investigation, project administration, writing – review and editing, software, resources. **Rosane M. M. Silva:** visualization, writing – review and editing. **Ana L. Penna:** formal analysis, visualization, writing – review and editing. **Gabrielle Oliveira:** visualization, writing – review and editing. **Aisha K. Yousafzai:** supervision, methodology, visualization, writing – review and editing. **Débora F. Mello:** conceptualization, supervision, methodology, visualization, writing – review and editing.

## Ethics Statement

This study received approval from the Research Ethics Committee of Ribeirao Preto Nursing School at the University of Sao Paulo (ethical appreciation number 5.951.090, 2023).

## Conflicts of Interest

The authors declare no conflicts of interest.

## Data Availability

The data supporting the findings of this study are available from the corresponding author upon reasonable request.
